# Ordinal SuStaIn: Subtype and Stage Inference for Clinical Scores, Visual Ratings, and Other Ordinal Data

**DOI:** 10.3389/frai.2021.613261

**Published:** 2021-08-12

**Authors:** Alexandra L. Young, Jacob W. Vogel, Leon M. Aksman, Peter A. Wijeratne, Arman Eshaghi, Neil P. Oxtoby, Steven C. R. Williams, Daniel C. Alexander, for the Alzheimer’s Disease Neuroimaging Initiative

**Affiliations:** ^1^ Department of Neuroimaging, Institute of Psychiatry, Psychology and Neuroscience, King’s College London, London, United Kingdom; ^2^ Centre for Medical Image Computing, University College London, London, United Kingdom; ^3^ Department of Computer Science, University College London, London, United Kingdom; ^4^ Department of Psychiatry, University of Pennsylvania, Philadelphia, PA, Unites States; ^5^ Center for Biomedical Image Computing and Analytics, University of Pennsylvania, Philadelphia, PA, Unites States; ^6^ Stevens Neuroimaging and Informatics Institute, Keck School of Medicine, University of Southern California, Los Angeles, CA, Unites States; ^7^ Queen Square Multiple Sclerosis Centre, Department of Neuroinflammation, Faculty of Brain Sciences, UCL Queen Square Institute of Neurology, University College London, London, United Kingdom

**Keywords:** subtyping, staging, Alzheimer’s disease, disease progression modelling, ordinal data

## Abstract

Subtype and Stage Inference (SuStaIn) is an unsupervised learning algorithm that uniquely enables the identification of subgroups of individuals with distinct pseudo-temporal disease progression patterns from cross-sectional datasets. SuStaIn has been used to identify data-driven subgroups and perform patient stratification in neurodegenerative diseases and in lung diseases from continuous biomarker measurements predominantly obtained from imaging. However, the SuStaIn algorithm is not currently applicable to discrete ordinal data, such as visual ratings of images, neuropathological ratings, and clinical and neuropsychological test scores, restricting the applicability of SuStaIn to a narrower range of settings. Here we propose ‘Ordinal SuStaIn’, an ordinal version of the SuStaIn algorithm that uses a scored events model of disease progression to enable the application of SuStaIn to ordinal data. We demonstrate the validity of Ordinal SuStaIn by benchmarking the performance of the algorithm on simulated data. We further demonstrate that Ordinal SuStaIn out-performs the existing continuous version of SuStaIn (Z-score SuStaIn) on discrete scored data, providing much more accurate subtype progression patterns, better subtyping and staging of individuals, and accurate uncertainty estimates. We then apply Ordinal SuStaIn to six different sub-scales of the Clinical Dementia Rating scale (CDR) using data from the Alzheimer’s disease Neuroimaging Initiative (ADNI) study to identify individuals with distinct patterns of functional decline. Using data from 819 ADNI1 participants we identified three distinct CDR subtype progression patterns, which were independently verified using data from 790 ADNI2 participants. Our results provide insight into patterns of decline in daily activities in Alzheimer’s disease and a mechanism for stratifying individuals into groups with difficulties in different domains. Ordinal SuStaIn is broadly applicable across different types of ratings data, including visual ratings from imaging, neuropathological ratings and clinical or behavioural ratings data.

## Introduction

Characterisation of disease progression patterns and heterogeneity among individuals can provide fundamental insights into the biology of a disease and is key to developing tools for patient stratification that can support precision medicine and healthcare. Disease progression models (Fonteijn et al., 2012; Jedynak et al., 2012; Donohue et al., 2014; Oxtoby et al., 2014; Young et al., 2014; Bilgel et al., 2016; Iturria-Medina et al., 2016; Schiratti, 2017; Koval et al., 2018; Li et al., 2018; Marinescu et al., 2019; Venkatraghavan et al., 2019; Firth et al., 2020) reconstruct the long-term temporal evolution of disease biomarkers from cross-sectional or short-term longitudinal data, enabling diagnosis, prognosis and stratification from biomarker measurements. In contrast to supervised machine learning techniques such as classification, which focus on a single disease stage, disease progression models infer fine-grained temporal patterns, providing the ability to generalise across disease stages and quantify disease trajectories in previously unseen detail. Disease progression models were primarily developed for use in Alzheimer’s disease, where the decades-long disease process prevents the collection of long-term datasets that span the full disease time course, but they are increasingly being applied in other neurodegenerative diseases, such as Multiple Sclerosis (Eshaghi et al., 2018) and Huntington’s disease (Wijeratne et al., 2018) and other long-term chronic conditions, such as respiratory diseases (Young et al., 2020b). However, the majority of disease progression modelling techniques rely on the assumption that all individuals follow a single common disease progression pattern, and so are unable to model disease subtypes which are prevalent in many diseases, and particularly in neurodegenerative diseases. Clustering identifies disease subgroups (Whitwell et al., 2009; Nettiksimmons et al., 2010, 2013, 2014; Noh et al., 2014; Racine et al., 2016; Zhang et al., 2016; Ferreira et al., 2020; Habes et al., 2020), providing new insights into disease heterogeneity, but lacks the ability to generalise across different disease stages, and so is unable to distinguish heterogeneity arising from differences in disease stage from heterogeneity due to the presence of disease subtypes.

The Subtype and Stage Inference (SuStaIn) algorithm (Young et al., 2018) allows disease progression modelling to be used in combination with clustering to identify subgroups of individuals with distinct disease trajectories. SuStaIn simultaneously clusters individuals into subgroups and characterises the trajectory that best defines each subgroup, thus capturing heterogeneity in both disease subtype and disease stage. The SuStaIn algorithm has been applied in a range of conditions including Alzheimer’s disease (Young et al., 2018; Aksman et al., 2020; Garcia et al., 2020; Vogel et al., 2021), frontotemporal dementia (Young et al., 2018; Young et al., 2020a), Multiple Sclerosis (Eshaghi et al., 2020) and Chronic Obstructive Pulmonary disease (Young et al., 2020b). From a mathematical perspective any disease progression model can be used in combination with SuStaIn, but in practice some disease progression models may be unfeasibly computationally intensive. Two disease progression models have been used with SuStaIn to date: the event-based model (Fonteijn et al., 2012; Young et al., 2014; Firth et al., 2020) and the piecewise linear z-score model (Young et al., 2018). The event-based model describes disease progression as a series of events, where each event corresponds to a new biomarker becoming abnormal. The piecewise linear z-score model describes disease progression as a series of stages, with each stage corresponding to a biomarker linearly increasing to a new z-score relative to a control population. The advantage of each of these two models is that they are not too computationally intensive and work with purely cross-sectional data, enabling SuStaIn to perform stratification based on a single visit.

As is the case with most disease progression models, the disease progression models used in combination with SuStaIn to date are designed to take continuous biomarker measurements as input, for example those derived from blood or fluid samples or medical imaging. Whilst continuous measures offer fine-scaled resolution and so can provide high precision, discrete ordinal data, such as visual ratings of images, neuropathological ratings, and clinical and neuropsychological test scores can provide unique and complementary information. Clinical and cognitive test scores, for example, are widely collected in clinical settings and directly measure skills and symptoms that affect an individual’s quality of life and reflect the severity of their disability. Meanwhile, neuropathological ratings offer direct measurement of disease pathologies, and thus can provide unique insights into the disease biology not possible with other techniques. Where imaging is used in a clinical setting, visual ratings of images are often already integrated into the clinical workflow, and thus can underpin diagnostic, prognostic and stratification tools that are more readily integrated into clinical practice. However, such measurements are not readily analysable by the majority of disease progression models, and neither of the disease progression models currently available for use with SuStaIn accommodate discrete ordinal data. The event-based model (Fonteijn et al., 2012; Young et al., 2014; Firth et al., 2020) doesn’t model different severity levels, instead assuming each event is a transition from ‘normal’ to ‘abnormal’. The piecewise linear z-score model (Young et al., 2018) doesn’t allow for discrete data as it describes continuous biomarker trajectories with gaussian noise. There is a need for the development of disease progression modelling techniques that can be used on discrete ordinal data to enable a broader range of analyses to be carried out on these data types, in line with the techniques already available for continuous data.

Here we introduce the scored events model, allowing SuStaIn to be used with ordinal data. The scored events model describes disease progression as a series of events, where each event corresponds to a biomarker transitioning to a new score. We term the resulting algorithm ‘Ordinal SuStaIn’. We verify the validity of Ordinal SuStaIn on simulated data, and that it out-performs the alternative option of using the existing piecewise linear z-score model (‘Z-score SuStaIn’) on ordinal data. We then demonstrate Ordinal SuStaIn by characterising heterogeneous trajectories of decline in subcategories of the Clinical Dementia Rating (CDR) scale.

## Materials and Methods

### The Scored Events Model

**FIGURE 1:**
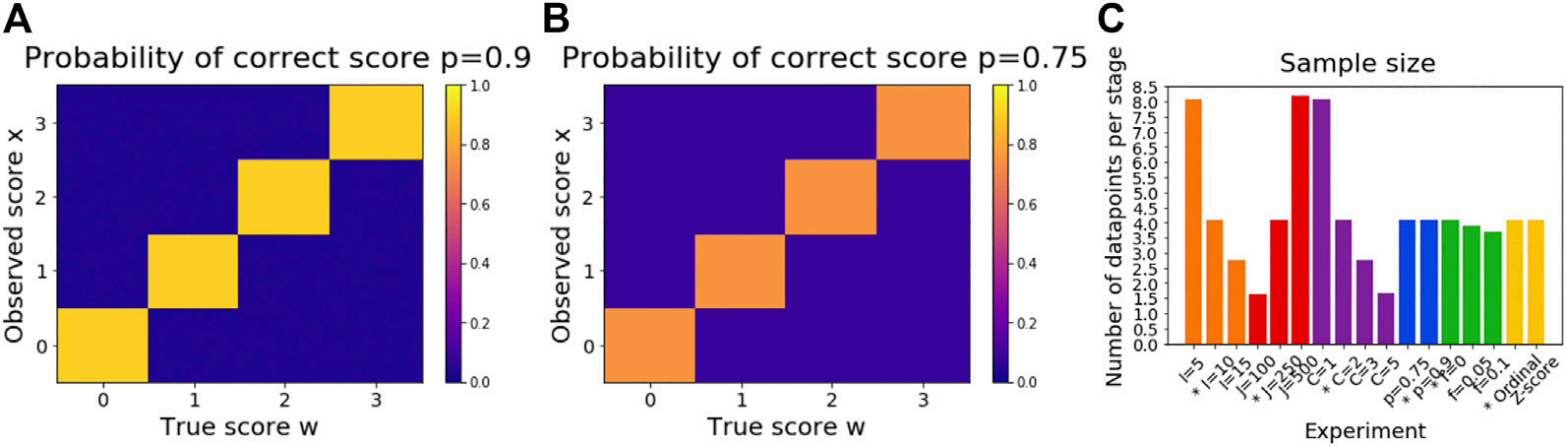
Illustration of simulation settings. Subfigure **(A)** shows $P\left(x_{ij}|E_{iw}\right)$ for the default proportion of correctly scored individuals *p* = 0.9. Subfigure **(B)** shows $P\left(x_{ij}|E_{iw}\right)$ for the setting *p* = 0.75. $P\left(x_{ij}|E_{iw}\right)$ can also be set to vary for each biomarker i and/or subject j. Subfigure **(C)** shows the expected number of datapoints for each stage of each subtype for each simulation setting.

We propose a scored events model to describe disease progression in Ordinal SuStaIn. The scored events model describes disease progression as a series of events, where each event corresponds to the transition of a biomarker to a new score. The occurrence of an event $E_{iw}$ in biomarker i for score w is informed by the measurements $x_{ij}\;$ of biomarker  i in subject j, where each biomarker has its own set of scores $w_{ir}\;=\;w_{i1}\;\ldots \;w_{iW_{i}}$, and starts from a minimum score $w_{i0}$. The whole data set $X\;=\;\left\{x_{ij}|i=1\ldots I,\;j=1\ldots J\right\}$ is the set of measurements of each biomarker in each subject. The most likely ordering of the scored events is the sequence S that maximises the data likelihood

$$P\;\left(X|S\right)=\overset{J}{\underset{j=1}{\prod }}\left[\sum_{k=0}^{K}P\left(k\right)\overset{I}{\underset{i=1}{\prod }}P\left(x_{ij}|E_{iw}\right)\right]\;,$$

where w=s(i,k) is the score reached by biomarker i at stage k in the sequence S; at stage 0, w = $w_{i0}$ for all biomarkers. The number of stages K is defined by the number of scored events included in the model, $K=1+\sum_{i=1}^{I}W_{i}$, i.e., the total number of scores included across all biomarkers. The form of the distribution $P\left(x_{ij}|E_{iw}\right)$ is fully flexible and can be chosen by the user. The scored events model simply takes as input the probability each datapoint has each score: for each measurement $x_{ij}$ of biomarker  i in subject j the user specifies the probability $P\left(x_{ij}|E_{iw}\right)$ that the ‘true’ score of measurement $x_{ij}$ is $E_{iw}$ for each score w as a matrix with dimensions $\;J\times W_{i}$ for each biomarker i. Here we use a categorical distribution (see Figure 1 for a visualisation) where

$$P\left(x_{ij}\text{|}E_{iw}\right)=\;\{\;\begin{array}{c} \;p\;\;\;\;\;\;\;\;\;\;\;\;\;\;\;\;\;if\;x_{ij}=w\\ \frac{1-p}{W_{i}}\;\;\;\;\;\;\;\;\;if\;x_{ij}\neq w \end{array}$$

thus p indicates the proportion of correctly scored individuals for each biomarker, and all other scores are assumed to be equally probable.

### Ordinal SuStaIn

The SuStaIn algorithm (Young et al., 2018) assumes a dataset consists of c clusters of individuals (subtypes) that undergo a common disease progression pattern, $S_{c}$. Each individual is a sample of an unknown subtype c at an unknown stage k along the disease progression pattern for that subtype. SuStaIn simultaneously optimises subtype membership and subtype progression patterns (which describe the stages of the disease). SuStaIn fits an increasing number of clusters up to a user-defined maximum, using Markov Chain Monte Carlo (MCMC) sampling to obtain samples of the progression pattern for each subtype, providing an estimate of the posterior distribution of each subtype progression pattern. Information criterion can be used to choose the optimal number of clusters by evaluating the number of clusters that best balances accuracy and complexity, such as the Cross-Validation Information Criterion used in (Young et al., 2018). Our proposed Ordinal SuStaIn algorithm uses the scored events model detailed above to describe the evolution of biomarkers at different stages. To this end, Ordinal SuStaIn uses the same implementation of the SuStaIn algorithm as in (Young et al., 2018), but replaces the data likelihood $P\left(X|S_{c}\right)$ for each subtype c with that of the scored events model described above.

### Simulated Data

We generated a series of simulated datasets to test the performance of Ordinal SuStaIn. To generate each dataset we randomly chose C subtype progression patterns, each described by a sequence S in which a set of scored events occur. We fixed the expected proportion $\pi_{c}$ of individuals belonging to each subtype c to be

$$\pi_{c}=\frac{C-c+2}{\sum_{c=1}^{C}\left[C-c+2\right]},$$

or equivalently,

$$\pi_{C-c+1}=\frac{c+1}{\sum_{c=1}^{C}\left[c+1\right]},$$

such that the proportion of individuals in each subtype decreased from the most prevalent subtype c=1 to the least prevalent subtype c=C. We then randomly assigned j=1…J  individuals to c=1…C  subtypes and k=0…K stages, using a weighted random sampling of subtype membership $c_{j}$ based on the proportion $\pi_{c}$ of individuals belonging to each subtype, and a uniform random sampling of stage $k_{j}$. The set of expected biomarker scores for each individual $\bar{W_{j}}=\left\{w_{i}\;\forall \;i\;where\;w_{i}=s\left(i,k_{j}\right)\;if\;k_{j}>0\;and\;{\text{w}}_{i}\;=\;w_{i0}\;if\;k_{j}=0\right\}$ was then evaluated and each individual’s biomarker data $\bar{X_{j}}$ was then sampled according to the categorical distribution $P\left(x_{ij}=w_{ij}\right)=p$ and $P\left(x_{ij}\neq w_{ij}\right)=\frac{1-p}{W_{i}}$, such that $P\left(x_{ij}|E_{iw}\right)$ follows the categorical distribution described above, as illustrated in Figures 1A,B. In our experiments we varied the number of biomarkers I, number of subjects J, number of subtypes C, proportion of correctly scored individuals p and a proportion of misdiagnosed individuals f who followed random subtype progression patterns not included in the simulated set of sequences S. By default we fixed the simulation settings to I=10, J=250, C=2, p=0.9, and f=0, varying each setting in turn to test settings of I=[5, 10, 15], J=[100, 250, 500], C=[1, 2, 3, 5], p=[0.75, 0.9], and f=[0, 0.05, 0.1]. We fixed the number of scored events to $W_{i}=3$ for all biomarkers i. Each experiment was performed three times for different randomly chosen subtype progression patterns and simulated datasets. The expected number of datapoints for each stage of each subtype varies across the different simulation settings, as illustrated in Figure 1C.

### Comparison With Z-Score SuStaIn

We performed one further simulation in which we used the default settings to generate simulated data but used Z-score SuStaIn rather than Ordinal SuStaIn to estimate the subtype progression patterns and subtypes and stages of individuals. Z-score SuStaIn uses a piecewise linear z-score model of disease progression, which describes disease progression as a series of events, where each event corresponds to a biomarker reaching a new z-score relative to a control population. The data in the control population is assumed to be normally distributed and the data is z-scored using this control population such that the control population has a mean of 0 and standard deviation of 1. In the piecewise linear z-score model, the biomarkers start at 0 (at stage 0), accumulating linearly between z-score events (each of which corresponds to a new stage) and accumulate to a final maximum z-score (reached at the last stage). The z-score events and the maximum z-score are specified by the user. To apply Z-score SuStaIn we z-scored the data using a control population consisting of individuals assigned to stage 0 in each experiment. The z-score events in Z-score SuStaIn were set to be the same as those in Ordinal SuStaIn by z-score transforming the score corresponding to each scored event. The maximum z-score was set to be the same as the maximum score of the scored event model by z-score transforming the maximum scores.

### Performance Evaluation: Progression Pattern Estimation

We estimated the most probable progression pattern ${\bar{S}}_{c}$ from the MCMC samples of the progression pattern by ordering the scored events according to their mean position in the sequence across samples. We measured the accuracy of the subtype progression patterns by calculating the average Kendall rank correlation τ (Kendall, 1945) between the most probable subtype progression patterns ${\bar{S}}_{c}$ estimated by SuStaIn and the ground truth subtype progression patterns ${\hat{S}}_{c}$ in each simulation. This is computed as

$$\tau =\;\frac{P-Q}{\sqrt{\left(P+Q+T\right)\left(P+Q+U\right)}}\;,$$

where P is the number of concordant pairs, Q is the number of discordant pairs, T is the number of ties in ${\bar{S}}_{c}$, and U is the number of ties in ${\hat{S}}_{c}$. Correspondence between the ground truth and simulated subtypes was achieved by matching each simulated subtype progression pattern ${\bar{S}}_{c}$ with the most similar ground truth subtype progression pattern ${\hat{S}}_{c}$. In nearly all experiments this was equivalent to matching the ground truth and simulated subtype progression patterns based on the proportion of individuals belonging to each subtype. The exception was for experiments with C=5 subtypes in which the fraction would sometimes be swapped between subtypes of similar sizes, and so matching the subtype progression patterns based on their correspondence with the ground truth ensured that correspondence was achieved between subtypes of similar sizes. We estimated the confidence in the position assigned to each scored event by evaluating the proportion of MCMC samples in which each scored event appeared in the same position as in the most probable progression pattern. We evaluated the accuracy of the confidence estimate by determining whether the ground truth position of each scored event fell within the 95% confidence estimates output by SuStaIn. To do this we tested whether the ground truth position of each scored event was within two standard deviations of the estimated mean position of each scored event across MCMC samples.

### Performance Evaluation: Subtyping and Staging

We computed the probability each individual belonged to each subtype and stage by computing the probability they belonged to each subtype (summed over stage) and stage (summed over subtype) for each MCMC sample and then averaging over MCMC samples, thus taking into account the uncertainty in the progression pattern of each subtype. We then assigned each individual to their most probable subtype and most probable stage. We estimated the confidence of the subtype and stage assignments by evaluating the probability of the subtype and stage that each individual had been assigned to. We evaluated the accuracy of the confidence estimates by determining whether the ground truth subtype and stage of each individual fell within the 95% confidence estimates output by SuStaIn. To do this we tested whether the ground truth subtype of each individual was assigned an average probability of at least 0.05, and whether the ground truth stage of each individual had a cumulative probability of more than 0.025 and less than 0.975.

### Performance Evaluation: Number of Subtypes

When comparing the estimated subtype progression patterns and subtype and stage assignments with the ground truth, we fixed the number of subtypes to be the same as the ground truth number of subtypes to enable a direct comparison. To give an indication of the accuracy of the number of subtypes estimated by SuStaIn we fitted up to C+1 subtypes in each experiment. We then evaluated whether the 95% confidence intervals of the overall model likelihood (obtained from the MCMC samples of the model likelihood) for the ground truth number of subtypes C overlapped with the 95% confidence intervals of the overall model likelihood for one less (C−1) subtype and one more (C+1) subtype than the ground truth number of subtypes. We considered SuStaIn to underestimate the number of subtypes if the 95% confidence intervals of the C subtypes model likelihood overlapped the confidence intervals for C−1 subtypes, or if the average model likelihood was greater for C−1 subtypes. We considered SuStaIn to overestimate the number of subtypes if the average model likelihood was greater for C+1 subtypes than C subtypes, and the 95% confidence intervals of the model likelihood for C+1 subtypes didn’t overlap the confidence intervals for C subtypes.

### Alzheimer’s Disease Neuroimaging Initiative Data

Data used in the preparation of this article were obtained from the Alzheimer’s Disease Neuroimaging Initiative (ADNI) database (http://adni.loni.usc.edu). The ADNI was launched in 2003 by the National Institute on Aging (NIA), the National Institute of Biomedical Imaging and Bioengineering (NIBIB), the Food and Drug Administration (FDA), private pharmaceutical companies and non-profit organisations, as a $60 million, 5 years public-private partnership. For up-to-date information, see http://www.adni-info.org. Written consent was obtained from all participants, and the study was approved by the Institutional Review Board at each participating institution.

CDR sub-scores (Hughes et al., 1982; Morris, 1993) from 819 participants in ADNI1 and 790 participants in ADNI2 were collated to obtain two independent datasets measuring sub-scores of memory, orientation, judgement, community, home and personal care. Each CDR sub-score can be assigned a score of 0 (no impairment), 0.5 (questionable impairment), 1 (mild impairment), 2 (moderate impairment) or 3 (severe impairment). Of the 819 ADNI1 participants, 229 were cognitively normal, 397 had mild cognitive impairment and 193 had a dementia diagnosis. Of the 790 ADNI2 participants, 293 were cognitively normal, 349 had mild cognitive impairment and 148 had a dementia diagnosis. We further collated follow-up CDR sub-scores at 6, 12, 18, 24 and 36 months follow-up visits to test the longitudinal consistency of the subtypes and stages assigned by Ordinal SuStaIn.

We ran Ordinal SuStaIn separately on baseline data from each of the ADNI1 and ADNI2 studies to obtain two independent estimates of CDR subtype progression patterns. We set the proportion p in $P\left(x_{ij}\text{|}E_{iw}\right)$ with an accurate score to 0.75 for each sub-score, based on the inter-rater reliability of CDR scores in the literature (Schafer et al., 2004). None of the ADNI participants had a score of three on any CDR sub-scale and so this score was excluded from the scored events model. We selected the optimal number of subtypes by performing three-fold cross-validation in each dataset and evaluating the Cross-Validation Information Criterion (Gelman et al., 2014; Young et al., 2018).

Individuals were assigned to subtypes and stages at baseline and at follow-up visits using Ordinal SuStaIn, with the subtyping and staging being performed independently in each dataset (i.e., using the subtype progression patterns estimated from the baseline data in each dataset separately). Subtypes were considered to be longitudinally consistent between a pair of visits if both visits were labelled as the same subtype. Stages were considered to be longitudinally consistent between a pair of visits if the stage either remained the same or increased at the later of the two visits.

## Results

### Simulated Data: Progression Pattern

**FIGURE 2:**
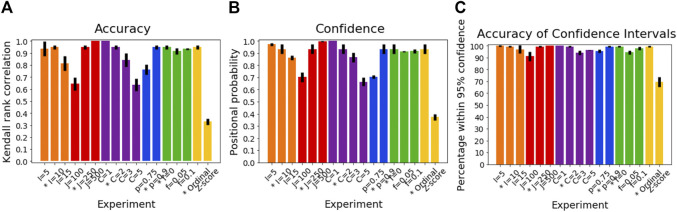
Performance of SuStaIn for recovering progression patterns. **(A)** Accuracy of Ordinal SuStaIn for recovering the ground truth subtype progression patterns, **(B)** the confidence SuStaIn assigned to the estimated subtype progression patterns, and **(C)** the accuracy of the confidence intervals SuStaIn assigned to the estimated subtype progression patterns. The x-axis shows the experiments in which we varied the simulated number of biomarkers I (orange), number of subjects J (red), number of subtypes C (purple), proportion p with an accurate score (i.e., the categorical probability each test score is accurate; blue), the proportion f of misdiagnosis (i.e., the proportion of individuals that follow randomly chosen alternative progression patterns; green), and the choice of algorithm (either the proposed Ordinal SuStaIn algorithm or the existing Z-score SuStaIn algorithm). The default value for each simulation setting is indicated with an asterisk on the x-axis.

Figure 2A shows the accuracy of SuStaIn for estimating subtype progression patterns under different simulation settings. In general, Ordinal SuStaIn gave a good accuracy across all settings, with a Kendall rank correlation between the estimated subtype progressions and the ground truth of >0.63 for all settings. When comparing Ordinal SuStaIn and Z-score SuStaIn under the default settings, the Kendall rank correlation using Z-score SuStaIn was only 0.33, compared to 0.95 for Ordinal SuStaIn. The confidence estimates of the position of each scored event provided by Ordinal SuStaIn (Figure 2B) gave a good indication of the true accuracy of the estimated progression patterns measured against the ground truth (Figure 2B reflects the trend in Figure 2A). Likewise, Figure 2C shows that the ground truth position of each scored event was generally within the 95% confidence intervals estimated by Ordinal SuStaIn for at least 95% of scored events (minimum of 94%, maximum of 100%). The confidence intervals obtained using Z-score SuStaIn were much less accurate with only 69% of the ground truth positions of the scored events being within the 95% confidence intervals estimated by Z-score SuStaIn.

**FIGURE 3:**
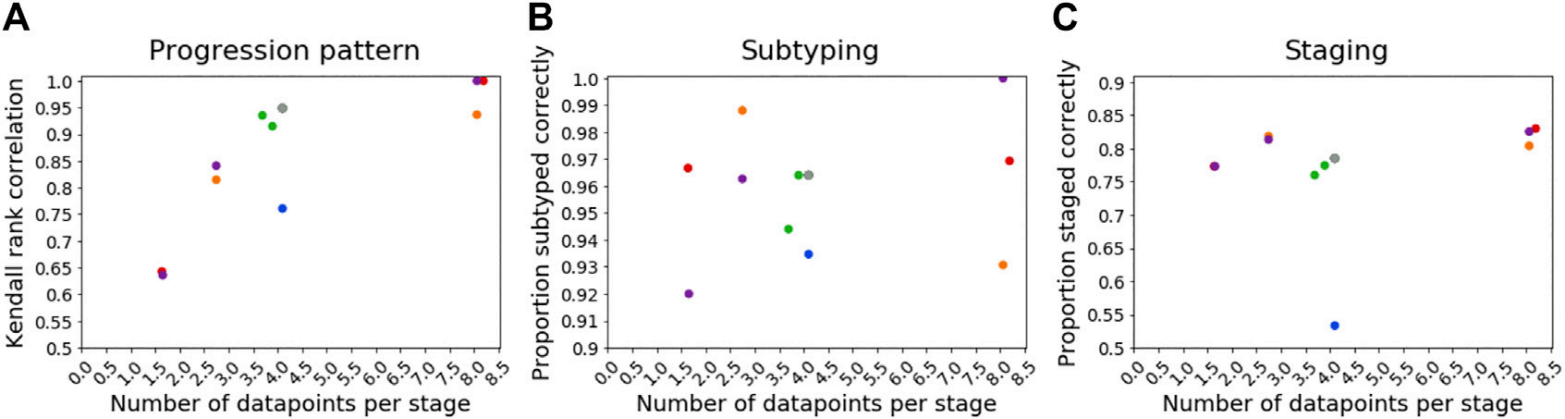
Relationship between sample size and accuracy**.** Each subfigure shows a scatter plot comparing the expected number of datapoints per stage for each simulation and the accuracy of Ordinal SuStaIn for **(A)** estimating subtype progression patterns, **(B)** subtyping individuals, and **(C)** staging individuals. Each simulation setting is plotted using the same colours used in Figures 2, 4, 5, except the default setting, which is shown in grey. The simulation using Z-score SuStaIn was excluded from these figures.

The Kendall rank correlation between the estimated progression patterns and the ground truth varied substantially with different simulation settings. The Kendall rank correlation decreased substantially when the number of biomarkers was set to I = 15 compared with I = 5 and I = 10, when the number of subjects was set to J = 100 rather than J = 250 and J = 500, when the number of clusters was set to C = 3 or C = 5 rather than C = 1 or C = 2, and when the proportion of correctly scored individuals was set to *p* = 0.75 compared to *p* = 0.9. As shown in Figure 3A, increasing the number of biomarkers, decreasing the number of subjects and increasing the number of clusters all reduce the number of datapoints per subtype and stage combination, with this decrease in sample size correlating with the decrease in the accuracy of the progression pattern. Figure 3A also shows that decreasing the proportion p of individuals that are scored correctly from *p* = 0.9 to *p* = 0.75, which makes the data noisier, further decreases the accuracy of the estimated progression patterns in addition to the effect of sample size.

### Simulated Data: Subtyping

**FIGURE 4:**
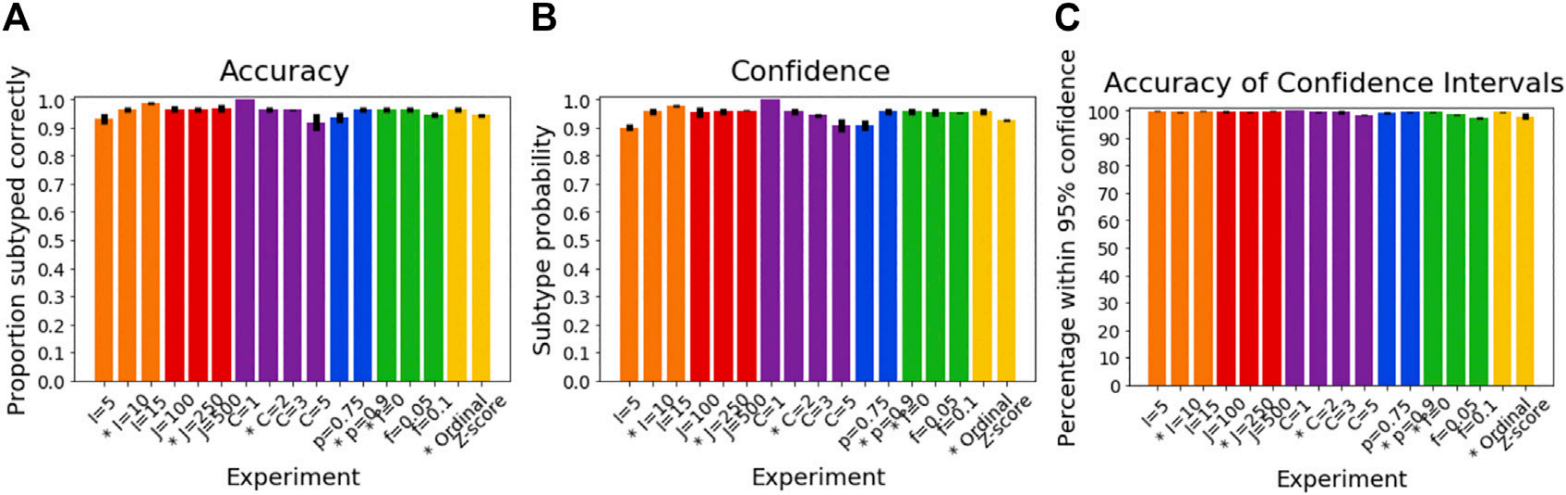
Performance of SuStaIn for subtyping individuals.** (A)** Accuracy of Ordinal SuStaIn for recovering the ground truth subtypes of individuals, **(B)** the confidence SuStaIn assigned to the estimated subtypes, and **(C)** the accuracy of the confidence intervals SuStaIn assigned to the estimated subtypes. As in Figure 2, the x-axis shows the experiments in which we varied the simulated number of biomarkers I (orange), number of subjects J (red), number of subtypes C (purple), proportion p with an accurate score (i.e., the categorical probability each test score is accurate; blue), the proportion f of misdiagnosis (i.e., the proportion of individuals that follow randomly chosen alternative progression patterns; green), and the choice of algorithm (either the proposed Ordinal SuStaIn algorithm or the existing Z-score SuStaIn algorithm). The default value for each simulation setting is indicated with an asterisk on the x-axis.

Figure 4A shows how the accuracy of SuStaIn for subtyping individuals varies with different simulation settings. SuStaIn was able to subtype individuals with high accuracy, with more than 92% of individuals being subtyped correctly for all simulation settings. Figure 4B shows that the confidence was a good reflection of the subtyping accuracy, following the same trend as Figure 4A. Figure 4C shows that all simulation settings gave 95% confidence intervals that were correct in at least 95% of subjects (minimum of 97%, maximum 100%). Figure 3B shows that the accuracy of the subtyping was not particularly related to the sample size. However, the sample size does remain reasonably large for each subtype across all simulation settings: the last subtype in the C = 5 experiment was the smallest, but still had an expected sample size of 25 subjects.

### Simulated Data: Staging

**FIGURE 5:**
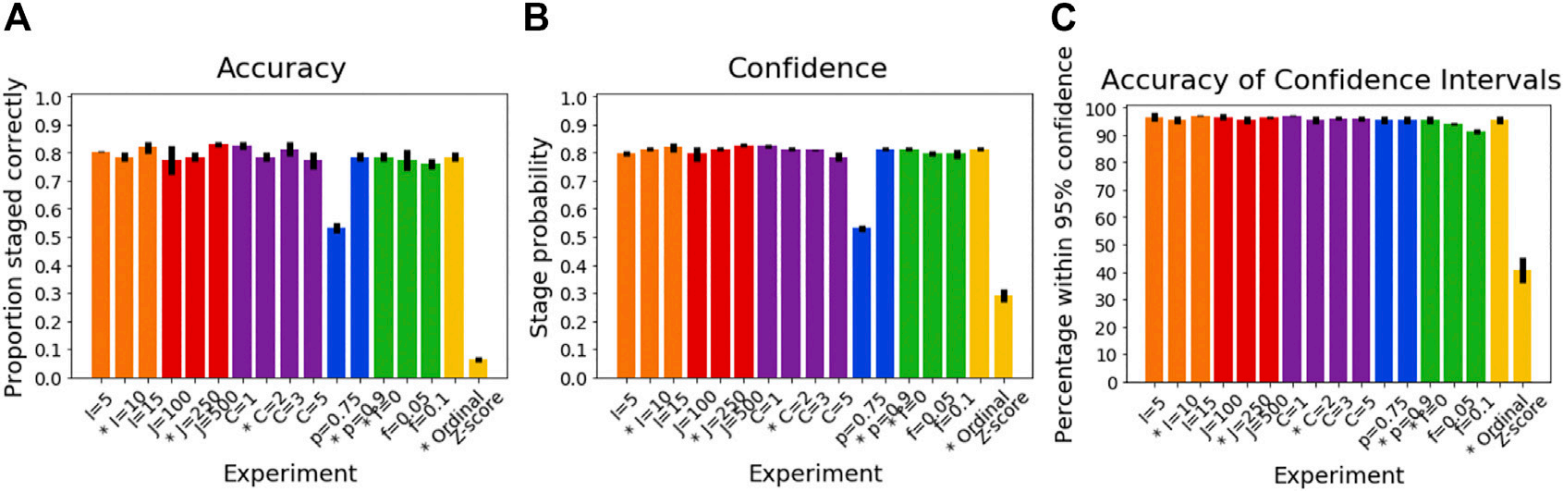
Performance of SuStaIn for staging individuals. **(A)** Accuracy of Ordinal SuStaIn for recovering the ground truth stages of individuals, **(B)** the confidence SuStaIn assigned to the estimated stages, and **(C)** the accuracy of the confidence intervals SuStaIn assigned to the estimated stages. As in Figures 2, 3, the x-axis shows the experiments in which we varied the simulated number of biomarkers I (orange), number of subjects J (red), number of subtypes C (purple), proportion p with an accurate score (i.e., the categorical probability each test score is accurate; blue), the proportion f of misdiagnosis (i.e., the proportion of individuals that follow randomly chosen alternative progression patterns; green), and the choice of algorithm (either the proposed Ordinal SuStaIn algorithm or the existing Z-score SuStaIn algorithm). The default value for each simulation setting is indicated with an asterisk on the x-axis.

**FIGURE 6:**
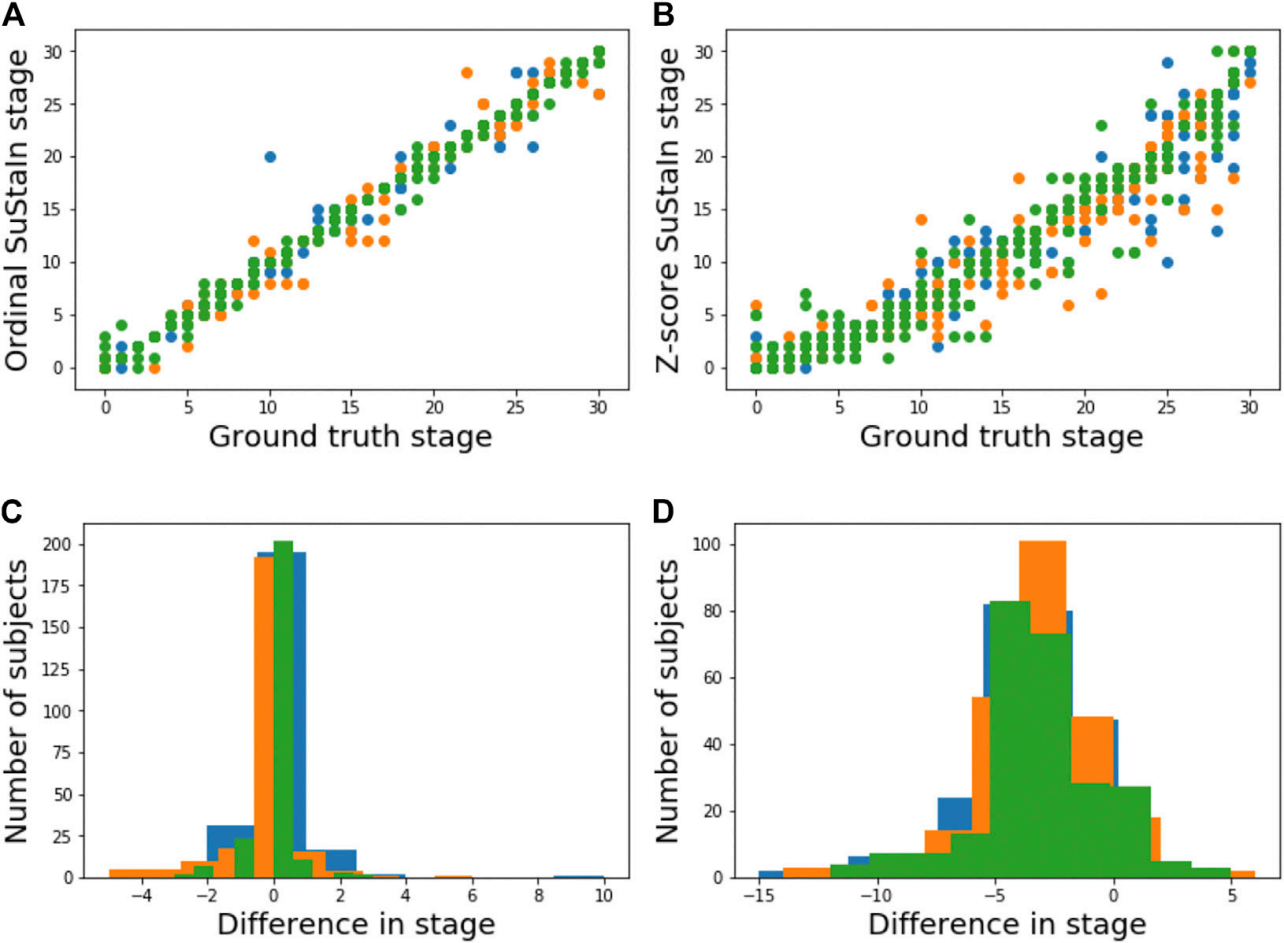
Comparison of staging performance using Ordinal SuStaIn and Z-score SuStaIn**.** The top row shows scatter plots comparing the ground truth stage in simulation and the estimated SuStaIn stage obtained from **(A)** Ordinal SuStaIn and **(B)** Z-score SuStaIn across three simulations (shown in different colours) performed using the default settings. The bottom row shows histograms of the difference between the ground truth stage and the stage estimated by **(C)** Ordinal SuStaIn and **(D)** Z-score SuStaIn across the three simulations.

Figure 5A shows the accuracy of the SuStaIn stages of individuals for different simulation settings. The SuStaIn stages were around 80% accurate for most simulation settings. There were two notable exceptions. The first was when the proportion of correctly scored individuals was set to *p* = 0.75, introducing more noise in the data and reducing the staging accuracy to 53%. The second was when Z-score SuStaIn was used rather than Ordinal SuStaIn, which staged only 6% of individuals correctly. Figure 5B shows that Z-score SuStaIn also has a lower confidence in the stages assigned to each individual, but that the stages are not within the 95% confidence interval estimated by Z-score SuStaIn, with only 40% of individual’s stages falling within the 95% confidence interval. For all other settings the confidence assigned by SuStaIn was a good reflection of the accuracy of the stages (Figure 5B follows the same trend as Figure 5A), and the confidence intervals were a good reflection of the confidence in each individuals stage assignment (Figure 5C), with at least the expected 95% of individuals ground truth stages falling within the 95% confidence intervals estimated by SuStaIn (minimum of 91% and maximum of 97%). Figure 3C shows that the staging accuracy increases slightly with sample size, but that the effect of noisy data (reducing the proportion of correctly scored individuals from *p* = 0.9 to *p* = 0.75) is much greater. Figure 6 shows the relationship between the ground truth stage and the stage assigned by Z-score SuStaIn. Z-score SuStaIn systematically underestimates the stage of each individual, as well as being less accurate than Ordinal SuStaIn.

### Simulated Data: Number of Subtypes

The number of subtypes was estimated accurately for all simulation settings, except when a proportion of misdiagnosed individuals f were included, or when Z-score SuStaIn was used instead of Ordinal SuStaIn. For f = 0.05, SuStaIn over-estimated the number of subtypes in two out of three experiments and for f = 0.10, SuStaIn over-estimated the number of subtypes in all three experiments. Z-score SuStaIn over-estimated the number of subtypes in two out of three experiments.

### Application to Clinical Dementia Rating Sub-scores

**FIGURE 7:**
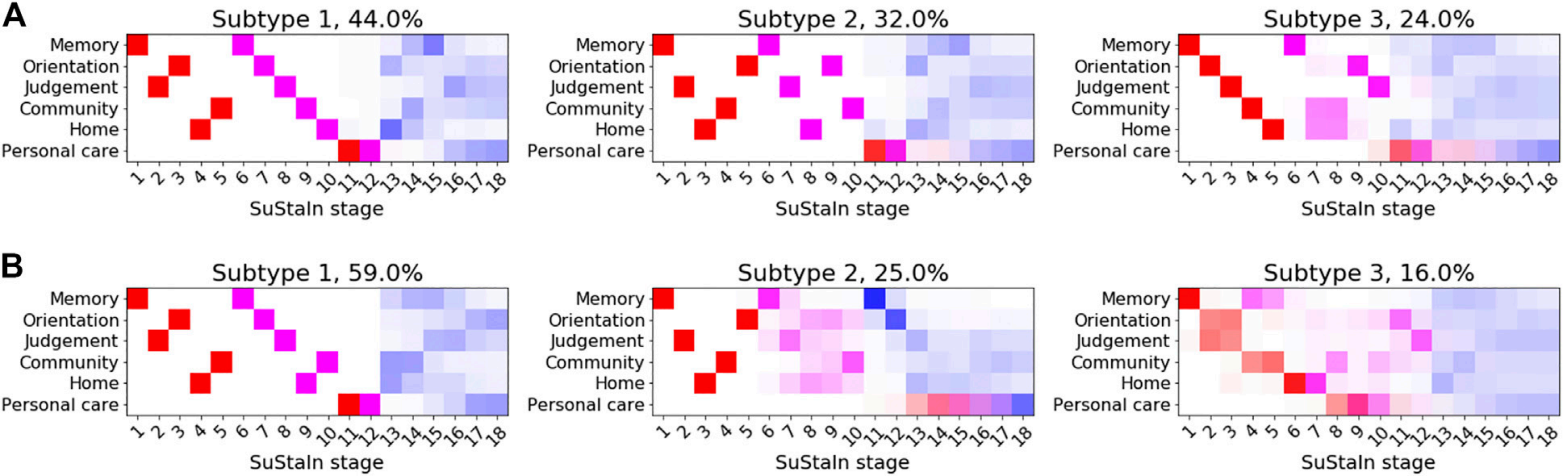
Clinical subtypes of Alzheimer’s disease based on CDR sub-scores. Subtypes of CDR ratings subtypes identified by applying Ordinal SuStaIn to **(A)** ADNI1 and **(B)** ADNI2. Each entry in the diagram represents the proportion of MCMC samples in which a particular scored event appears at a particular position along the progression pattern, with CDR = 0.5 shown in red, CDR = 1 in magenta and CDR = 2 in blue.

Figure 7 shows the subtype progression patterns estimated from applying Ordinal SuStaIn to CDR sub-scores in ADNI1 and ADNI2 separately. Three subtypes with distinct progression patterns were identified independently in each dataset, which we describe as 1) ‘typical’—the most numerous group, with memory problems at early SuStaIn stages, followed by difficulties with orientation and judgement and problem solving, and then difficulties with home life and community affairs, 2) ‘orientation-spared’—remaining relatively well-oriented until later SuStaIn stages, and 3) ‘outliers’—not following the ‘typical’ or ‘orientation-spared’ CDR sub-score progression pattern. The progression patterns were consistent between the two datasets, supporting the existence of three Alzheimer’s subgroups with distinct clinical progression.

### Subtyping and Staging Using Clinical Dementia Rating Sub-scores

**FIGURE 8:**
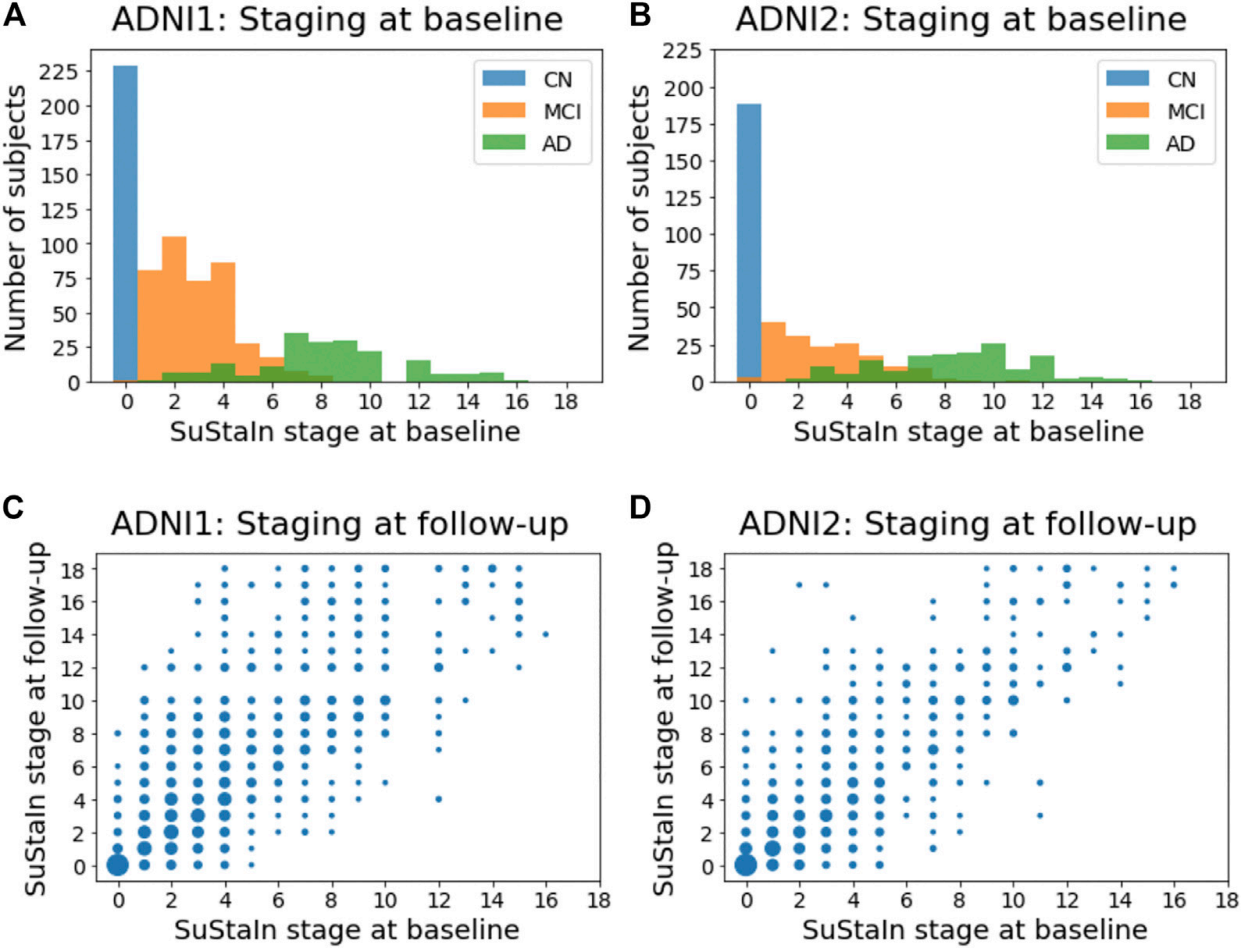
Staging individuals using CDR sub-scores. The top row shows histograms of the SuStaIn stages of individuals in **(A)** ADNI1 and **(B)** ADNI2. The bottom row shows scatter plots comparing the SuStaIn stages of individuals at baseline and follow-up in **(C)** ADNI1 and **(D)** ADNI2. The size of each point represents the number of individuals. CN = cognitively normal; MCI = mild cognirive impairment; AD = Alzheimer’s dsease.

Figures 8A,B show the distribution of the stages assigned to individuals by Ordinal SuStaIn at the baseline visit in ADNI1 and ADNI2. As expected, cognitively normal individuals had the lowest stages, followed by individuals with mild cognitive impairment, whilst individuals with Alzheimer’s disease had the highest stages. There was a clear separation between cognitively normal and Alzheimer’s disease subjects, with all cognitively normal subjects being assigned to stage 0 and all Alzheimer’s disease subjects being assigned to stage 2 or above. Figures 8C,D compare the stages assigned to individuals by Ordinal SuStaIn at baseline and at follow-up. The follow-up visits were generally longitudinally consistent, i.e., at follow-up individuals either remained at the same stage or advanced in stage compared to baseline. In ADNI 1, 2113 of 2456 follow-up visits (86%) were longitudinally consistent, and in ADNI2, 1606 of 1885 follow-up visits (85%) were longitudinally consistent.

The subtypes assigned to individuals by Ordinal SuStaIn generally remained consistent at follow-up visits. Assigning individuals to subtypes using CDR scores is difficult as several of the stages are predicted to give the same CDR values across more than one subtype. For example, at stage 5 of all subtypes, CDR values are predicted to be 0 for the personal care rating and 0.5 for all the other sub-scales. Likewise, at stage 6 of all subtypes, CDR values are predicted to be 0 for the personal care rating, one for the memory score, and 0.5 for all the other sub-scales. Naively comparing each pair of visits that had CDR scores available at both visits (excluding individuals assigned to SuStaIn stage 0 at either visit and therefore unable to be subtyped), we found that the same subtype was assigned at both visits in 3,017 of 5,129 pairs of visits (59%) from ADNI1, and 2035 of 2,728 pairs of visits (75%) from ADNI2. Performing the same analysis but instead considering only individuals confidently assigned to subtypes (probability greater than or equal to 0.75), and thus removing individuals who were at stages where the subtypes are indistinguishable, we found that the same subtype was assigned at both visits in 143 of 190 pairs of visits (75%) from ADNI1, and in 157 of 169 pairs of visits (93%) from ADNI2.

## Discussion

In this study we developed Ordinal SuStaIn, an extension of the SuStaIn algorithm to allow SuStaIn to be used with discrete scored data. We demonstrated strong performance of Ordinal SuStaIn on simulated data and much better performance than using Z-score SuStaIn, which is designed for continuous data only. We applied Ordinal SuStaIn to CDR scores to identify three CDR subtypes that were longitudinally consistent and replicable across independent data from ADNI1 and ADNI2.

The simulation results highlight the scenarios in which Ordinal SuStaIn performs best. In particular, the progression patterns are more accurately estimated when the average number of data points is more than three per stage. However, the confidence estimates still provided accurate information about the range of possible progression patterns and subtypes and stages of individuals, regardless of the simulation setting. The accuracy of the progression patterns also does not hugely impact on the subtyping and staging accuracy. In general, noise in the data has the largest effect of all settings, adversely affecting the ability to estimate the progression patterns and the stages of individuals. We also found that the number of subtypes is likely to be overestimated when a proportion of misdiagnosed individuals are included in the dataset. Misdiagnosed individuals are typically grouped into an outlier cluster with no distinct progression pattern.

We therefore propose the following guidelines for using Ordinal SuStaIn:

• Report the uncertainty in the progression patterns and the subtypes and stages of individuals by showing the positional variance diagrams or other visual representations of the uncertainty.
• In cases where there is low confidence take uncertainty into account in any subsequent analysis and reporting of results by clearly presenting the caveat that there is low confidence in a particular progression pattern.
• Small clusters with high uncertainty (proportion of individuals belonging to the cluster less than 10% and high uncertainty in the progression patterns illustrated by the positional variance diagrams) in the progression pattern should be reported as possibly being groups of outliers rather than subtypes.
• Where possible choose datasets and scored events to have an average of more than three data points per stage.
• Where possible choose biomarkers with a good signal to noise ratio.

Ordinal SuStaIn requires the user to input the probability $P\left(x_{ij}|E_{iw}\right)$ that the ‘true’ score of measurement $x_{ij}$ is $E_{iw}$. This allows complete flexibility in the probability distributions of the scores, which can vary by biomarker, score, and even by individual if desired. This allows the user to model, for example, some scores being difficult to distinguish from one another, whilst others are easily distinguished, or individualised confidence ratings for each score. $P\left(x_{ij}|E_{iw}\right)$ would ideally be estimated by comparing assigned scores for each biomarker with a ground truth, in which the scorer is blinded to the ground truth score. In the absence of a ground truth, $P\left(x_{ij}|E_{iw}\right)$ can be approximated by looking at test-retest reliability.

Z-score SuStaIn performed poorly at estimating progression patterns and stages of individuals for discrete data. Z-score SuStaIn uses a piecewise linear z-score model, which assumes that each biomarker transitions linearly between scores. This alters the expected value of each biomarker at each stage, with the majority of stages modelling biomarker values that don’t exist in the data, leading to inaccuracy in the estimation of the subtype progression patterns and the stages of individuals. Z-score SuStaIn further assumes the errors on the data are normally distributed, which means that there are predicted to be more individuals with lower and higher scores than exist in the data. This causes a systematic overestimation of the stages of individuals at early stages and an underestimation of the stages of individuals at late stages. In this case the overall trend is to underestimate the stages of individuals as there are more stages representing scored events that have a positively skewed distribution than a negatively skewed distribution. Z-score SuStaIn also tends to overestimate the number of subtypes in the data to account for poor modelling of the subtype progression patterns.

Ordinal SuStaIn identified three clinical Alzheimer’s subgroups with distinct patterns of decline in CDR sub-scores. The subgroups were independently identified in ADNI1 and ADNI2 and the subtypes and stages were longitudinally consistent at follow-up visits taken over a 3 year time frame. These subgroups may simply illustrate different cognitive trajectories experienced by individuals, there may be different underlying biological disease processes (Mukherjee et al., 2018), or there may be a proportion of individuals with other neurodegenerative diseases or atypical variants (Scheltens et al., 2017). Further work will be required to validate these subtypes in a wider range of clinical settings, and to test whether the subtypes correspond to distinct biological subgroups.

There are now three forms of SuStaIn that can be used in different settings: the new Ordinal SuStaIn algorithm proposed here, Z-score SuStaIn and Event-based SuStaIn. Ordinal SuStaIn uses a scored events model to describe discrete scored data, Z-score SuStaIn uses a piecewise linear z-score model to describe continuous data with normally distributed noise, and Event-based SuStaIn uses an event-based model to describe discrete or continuous biomarkers transitioning from normal to abnormal. Future work will explore whether it is possible to develop an integrated version of SuStaIn that can allow different types of data to be modelled simultaneously. Extensions to model subtypes conditioned on different variables would also be a valuable addition, for example modelling how genetics, demographics, lifestyle factors, multi-morbidity, and electronic health records are related to subtype assignment or how subtype assignment alters the probability of different outcomes, such as developing a particular condition or long-term health outcomes. Another important avenue for future work is incorporating longitudinal data to estimate the time between different stages.

All forms of the SuStaIn algorithm rely on several assumptions to infer temporal subtype progression patterns from cross-sectional data. One assumption is that biomarker trajectories increase monotonically with disease progression, enabling identifiability of the progression patterns. This monotonicity assumption is made at the population level rather than at an individual level, which enables SuStaIn to allow for reversion in disease stage; individuals who revert will be assigned a lower stage at follow-up than at baseline. In future work there may be possibilities to relax this assumption by allowing a subset of biomarkers to be non-monotonic or incorporating longitudinal data to establish the time directionality. Another design choice in the SuStaIn algorithm is that the number of stages is fixed based on the number of biomarkers and scores. This simplifies the discrete optimisation procedure underlying SuStaIn by reducing the number of dimensions of the search space but can lead to redundant model complexity. Future versions will test whether it is possible to optimise the number of stages to enable more compact subtype progression patterns. However, under the current version of the SuStaIn algorithm, stages of a subtype progression pattern that are under-represented by samples can be identified by looking at the uncertainty in the positional variance diagrams. In addition, the model complexity can be reduced pre-emptively by limiting the number of features for small datasets, for example by using the rule of thumb described earlier of ensuring at least three subjects per stage. Another assumption that leads to redundancy in the subtype progression patterns is that each subtype progression pattern is unique; in fact, some subtypes may merge or split at some points in the progression. Future versions of the SuStaIn algorithm will explore whether merging and splitting of subtype progression patterns can be incorporated.

We proposed Ordinal SuStaIn, a variant of the SuStaIn algorithm for use with discrete scored data. We demonstrated that Ordinal SuStaIn out-performs available versions of SuStaIn in this setting and provides good performance in simulation. Ordinal SuStaIn is applicable to any discrete scored data. Here we applied Ordinal SuStaIn to CDR scores to reveal three distinct CDR subtypes in Alzheimer’s disease, however Ordinal SuStaIn is readily applicable to visual ratings data, such as from neuropathology or imaging, other clinical, neuropsychological or behavioural scores, and across a wide range of conditions, including other neurodegenerative diseases and respiratory diseases.

## Data Availability

The Alzheimer’s disease data used in this study are publicly available from the Alzheimer’s Disease Neuroimaging Initiative (ADNI) database (adni.loni.usc.edu). Source code for the Ordinal SuStaIn algorithm is available at https://github.com/ucl-pond/pySuStaIn together with a jupyter notebook enabling the simulations to be reproduced.
